# The importance of online childbirth preparation courses

**DOI:** 10.18332/ejm/185867

**Published:** 2024-04-09

**Authors:** Vicentia C. Harizopoulou, Evangelia Saranti, Angeliki Antonakou, Victoria Vivilaki

**Affiliations:** First Department of Obstetrics and Gynecology, Papageorgiou General Hospital, Thessaloniki, Greece; Department of Midwifery Science, School of Health Sciences, International Hellenic University, Thessaloniki, Greece; Department of Midwifery, School of Health and Care Sciences, University of West Attica, Athens, Greece

**Keywords:** childbirth, preparation, online courses

Online antenatal courses are educational programs conducted through various online platforms. They are facilitated mainly by qualified midwives who provide accurate and up-to-date information to the participants. The courses provide expectant parents with information, guidance, and support on childbirth, and the transition to parenthood that they would receive within traditional structures and courses^1^.

The courses typically cover various topics of interest, such as physiological changes, physical and emotional well-being during pregnancy, prenatal and postnatal care, information on labor and birth options, newborn care, and breastfeeding^2,3^. The content can be presented synchronously or asynchronously, in a closed or open group, and in various downloadable or not formats, including lectures, live webinars, discussion forums, videos, and interactive quizzes^4^.

Two are believed to be the key features of online antenatal courses: their flexibility and accessibility, with the latter emerging especially during the COVID-19 pandemic. Measures taken worldwide to ensure public health have limited the ability of pregnant women to attend in-person antenatal courses within traditional structures. Midwives worldwide, following that emerging need were motivated to convert in-person antenatal courses to innovative remote online sessions^1,5-8^. This extensive use of online antenatal education raised discussion on the method’s advantages, disadvantages, and potential benefits for specific groups of pregnant women. It is also worth noting that this discussion is taking place while implementation guidelines and research data on the effectiveness of online antenatal courses, compared to the traditional approach, are limited^1^.

## Advantages of online antenatal courses

There are many advantages of using an online platform to deliver antenatal courses. Firstly, participants value online antenatal classes’ time efficiency, convenience, flexibility, and accessibility^6,9-11^. They feel free to engage with the course material using their computer or mobile device anytime and from the comfort of their private space. They also feel that they save time and costs by eliminating the need for transportation. Moreover, this ease of access to online courses has a particular implementation in pregnant women with mobility issues and those living in remote or rural areas by breaking down the distance barrier and providing valuable information, guidance, and confidence in birth preparation^9,12^. In Greece, for example, there are approximately 6000 islands and islets. Of all these islands, around 250 are inhabited. At the same time, many high-altitude mountain villages are not easily accessible, especially during the winter months. Online antenatal courses benefit all those individuals who might not otherwise have access to prenatal preparation programs.

Secondly, online antenatal classes address the needs and expectations of millennial parents, who are online information seekers exposed to and comfortable with technology^2,10,13-15^. Therefore, the variety of resources (multimedia content, midwife-led sessions, interactive modules) an online class can offer can be tailored to the unique learning needs and characteristics of the woman^13^ by allowing her to revisit specific topics and having autonomy by selecting the content, the pace and the extent of involvement^6^. This is also linked to the recommendations concerning the use of the principles of adult education in antenatal education programs^6^.

Additionally, every implementation of online antenatal courses has the potential to reach a wider cohort of participants^9,16^. This feature enhances cost-effectiveness and the dynamic to be extended globally to an international population^9,16^. Antenatal education is not equally widespread around the globe. Online courses can provide an informative and consultative connection between women seeking antenatal education and midwives practicing in different countries. Furthermore, the supremacy of remote courses is evident in immigrant participants, who face multiple obstacles concerning prenatal care and education^17^. Choosing to have online antenatal courses by midwives from their country of origin helps them feel more culturally accepted and relaxed without worrying about language barriers. This choice will also provide pregnant immigrants with a connection with other expectant mothers from their country and an opportunity to build supportive networks.

Further advantages of online antenatal courses include the provision of anonymous identity to those feeling uncomfortable^9^, the avoidance of stigmatization^18^ or judgment^6^, and the more convenient participation of busy partners and other family members. The last-mentioned is essential, as pregnant women feel that antenatal courses enhance their partners’ understanding and support during pregnancy and childbirth, keeping them involved in the decision-making and the transition to parenthood^19^. Moreover, fathers’ participation in in-person or virtual antenatal classes improves their attachment to the infant^20^.

Finally, as the COVID-19 pandemic revealed, online antenatal courses are a good and safe alternative to in-person traditional courses during disasters and epidemics^21-23^. They support antenatal care, provide cutting-edge knowledge, and decrease prenatal distress and anxiety levels^23-25^.

## Disadvantages of online antenatal courses

While online antenatal courses have many advantages, there are also cautions of some potential disadvantages of exclusive online antenatal education. Pregnant women attending antenatal courses desire social interaction^2,11,13^ with people in the same situation, which offers the potential for future friendship and support, particularly at a community level^2,5,13,19^. Online courses usually lack direct communication, free-time interaction, and non-verbal cues^1^, factors closely linked to good communication and building peer support networks during antenatal and postnatal periods. Those factors also restrict the relationship and the inquired personalized guidance^1^ between the midwife and the woman. By participating in online courses, they lose chats in the interim of the classes, hallway discussions, natural social chemistry, and coffee talk, as educators vividly describe^1,24^.

Another drawback of online antenatal courses concerns hands-on demonstrations, embodied learning, and practical skills for labor and early parenting. Replicating and practicing birthing positions, breathing patterns, and massage techniques online are challenging and demanding^1^. In the same way, online courses without direct interaction and instant feedback can lead to misconception or misunderstanding if the participants have queries and need further explanation or in-depth analysis^15,19^. Therefore, pregnant women attending online antenatal courses desire access to educators and highlight the need for direction and support from them^6^.

Additional obstacles during online antenatal courses from the facilitator’s and educator’s part include the potential medico-legal risks raised, their insufficient familiarity with technological troubleshooting, the handling of what is called ‘Zoom fatigue’ and distractions, often due to participants’ ‘multitasking’ while attending the course^6,11,12,25^.

Moreover, according to the participants’ perspectives, a pregnant woman might be suspicious of sharing personal details in online courses and skeptical about the quality of the information and the expertise of the educators^9^. Pregnant women generally prefer professional and conventional sources of information as more trustworthy and valuable^15,19^.

Finally, a significant disadvantage of online antenatal courses is that not everyone may be comfortable with or have access to the necessary technology or reliable internet connection. Those technical challenges and internet literacy may cause discrimination and exclusion, especially for vulnerable pregnant women^6,24,26^.

## How should midwives move on?

Midwives support antenatal education and implement childbirth preparation courses to meet the needs and expectations of women, their families, and their communities. It is suggested that millennial women expect and appreciate an innovative approach to antenatal education^11^. They also need information and support in a way and through a channel that they can easily understand and access^27^. On the other hand, online platforms alter how women experience maternity services and their assessment and reception of information, which requires attention^13,28^. Several researchers underline the urge to redefine the midwife’s role as an antenatal educator in the digital era^1,11,29^. Most of them reasonably advocate for the maintenance of the core values and content of antenatal education, such as the continuity of care, the goal of woman-centeredness, trustworthiness, the empowering of women, the opportunities for social support and meaningful interaction^1,6,11,13,29^. Thus, recommendations and protocols for designing online antenatal education are critical and must be formulated on research data.

There is a need for high-quality, randomized trials with sufficient sample sizes to investigate and evaluate the effect of online antenatal education on women, their families, and communities^16^. Documented effects on specified primary and secondary pregnancy outcomes will stimulate the debate among midwives, stakeholders, and policy-makers, and indicate the future direction of online antenatal classes. Moreover, future research should specify the assumed differences between in-person and online antenatal courses regarding efficacy, knowledge, skills attained, and participants’ overall satisfaction^30^.

Finally, one significant dimension of providing online antenatal education is the educator’s acquisition of digital skills. Digital literacy should be enhanced in contemporary undergraduate midwifery programs to prepare the next generation of midwives for ‘digital care’ in the evolving digital space and establish the future professional framework^11,31^.

It will remain unclear whether online antenatal education would have been so widespread if it had not been proceeded by the COVID-19 pandemic. Antenatal education is undoubtedly essential in the transition to parenthood, and its online form is an emerging field with many advantages that should be noticed. Attention to the regulation of the online antenatal educator, the planning, the documentation of procedures, and the ongoing evaluation will facilitate future implementation, enhancement, and sustainability of online childbirth preparation courses.

## Data Availability

Data sharing is not applicable to this article as no new data were created.
